# Trends of *Toxoplasma gondii* and common transfusable venereal infections among blood donors in Menoufia Province, Egypt

**DOI:** 10.1038/s41598-024-70740-9

**Published:** 2024-09-09

**Authors:** Marwa A. Gouda, Sara A. Saied, Walaa Mohamed Omar Ashry, Raafat Abd-Rabow Abd-Eltwab, Mohamed Morshdy Aldesoky, Omnia Ahmed El-dydamoni, Marwa Yousef, Mona M. El-Derbawy

**Affiliations:** 1 Department of Clinical and Molecular Parasitology, National Liver Institute, Menoufia University, Menoufia, Egypt; 2 Department of Clinical Pathology, National Liver Institute, Menoufia University, Menoufia, Egypt; 3 Department of Medical Microbiology and Immunology, Damietta Faculty of Medicine (Girls), Al-Azhar University, Damietta, Egypt; 4 Department of Medical Microbiology and Immunology, Damietta Faculty of Medicine, Al-Azhar University, Damietta, Egypt; 5 Department of Medical Microbiology and Immunology, Faculty of Medicine for Girls (Cairo), Al-Azhar University, Cairo, Egypt; 6 Department of Epidemiology and Preventive Medicine, High Institute of Public Health, Alexandria University, Alexandria, Egypt; 7 Department of Medical Parasitology New Damietta Faculty of Medicine (Girls), Al-Azhar University, Damietta, Egypt

**Keywords:** *Toxoplasma gondii* Prevalence, Blood group antigens, Egypt, HBV, HCV, HIV, Microbiology, Epidemiology

## Abstract

Blood transfusion has a hazard of transmission of many pathogens, including *Toxoplasma gondii* (*T. gondii*) and other venereal infections. It is crucial to conduct epidemiological surveillance to detect the prevalence of these pathogens. The study aimed to assess the seroprevalence of *T. gondii* and common transfusable venereal infections among healthy blood donors in Menoufia Province, Egypt, and identify associated risk factors. Four hundred twenty individuals were recruited between January and April 2023 for cross-sectional descriptive research from the blood banks of Menoufia University medical hospitals. Collected blood samples were screened for anti-*T. gondii* IgM and IgG, HBsAg, anti-HCV antibodies, HIV p24 antigen and anti-HIV antibodies, and anti-*Treponema pallidum* antibodies. 46 (11.0%) and 22 donors (5.2%) individuals tested positive for anti-*T. gondii* IgG with a 95% CI (8.3–14.6) and IgM with a 95% CI (3.5–8.1), respectively, while one patient (0.2%) was positive for both antibodies. Regarding venereal infections, 12 (2.9%) were positive for HBV, 6 (1.4%) were positive for HCV, 7 (1.7%) were positive for HIV, and none of the tested population showed positivity for syphilis. Female gender, consumption of raw meat, agricultural environment, poor awareness about *T. gondii*, and blood group type (especially AB and O groups) were identified as independent risk factors for *T. gondii* infection. The study highlights the importance of testing blood donors for *T. gondii* and common transfusable venereal illnesses. Starting health education programs and preventative measures, such as suitable meat handling and cleanliness practices, is critical for minimizing the occurrence of these illnesses. Larger-scale additional study is advised to confirm these results and provide guidance for public health initiatives.

## Introduction

Blood transfusion is a critical medical procedure vital for patients’ treatment. Every year, millions of people are exposed to avoidable life-threatening risks as a result of hazardous blood transfusions. The major transfusion-transmitted infections are Hepatitis B virus (HBV), Hepatitis C virus (HCV), human immunodeficiency virus (HIV), and syphilis, which pose significant threats to recipient safety^1^.

*Toxoplasma gondii* is a food-borne zoonotic protozoan parasite capable of infecting all homoeothermic vertebrates; however, felids, which are members of the Felidae family, serve as the definitive hosts for (*T. gondii* ) infection, as both the sexual (intestinal) and asexual (tissue) cycles occur simultaneously in these animals (cats), resulting in un-sporulated non-infectious oocyst elimination and excretion^2^.

Oocysts may shed in vast numbers, even though they typically shed within 1–3 weeks. Oocysts sporulate in the environment in one to five days and spread infection. Warmer settings can facilitate sporulation more quickly, which increases the rate at which oocysts are found in the environment^3^. Temperature, humidity, and precipitation patterns all influence the survival and dissemination of *T. gondii* oocysts in the environment^4^. Warmer temperatures and greater rainfall can help oocysts survive and spread, potentially boosting infection rates in both animal and human populations^5^.

The infection with *T. gondii* usually appears as mild manifestations observed on exposure in immunocompetent people, such as warmth, tiredness, and cervical lymphadenopathy, which are self-limited; however, pneumonitis and encephalitis are complications of the infection, which is severe in immunocompromised people (such as AIDS patients) and blood recipients (such as those with thalassemia, haemophilia, dialysis patients, organ transplant recipients, and neonatal jaundice)^6,7^.

Co-infections can increase the severity of some infectious disorders. It has the potential to affect immune responses, and disease severity, and increase inflammatory cytokines^8^. Since *T. gondii* is considered one of the most successful parasites on the planet, the *T. gondii* disease burden has been classified as one of the most significant parasitic disorders. In order to reduce the occurrence of *T. gondii* infection among humans, it is urgent to understand the current status of this pathogen. Our study aimed to estimate the current situation of *T. gondii* and other transfusable venereal infections among blood donors in Menoufia Province, reflecting previously unknown regional outlines. Also, the study evaluated possible risk factors linked to *T. gondii* exposure in the population. Finally, the study intended to propose community-wide methods to raise awareness and prevent *T. gondii* infection.

## Subjects and methods

### Ethical approval and consent to participate

This study was conducted in accordance with the ethical principles outlined in the Declaration of Helsinki and was approved by the National Liver Disease Institute’s research ethics committee (NLI IRB procedure N. 00,422/2022). All subjects have given informed consent after being informed about the study’s objectives, the importance of participation as part of the community, and any potential adverse side effects of puncture. All subjects gave informed consent after being informed about the study’s objectives, the importance of participation as part of the community, and any potential negative side effects of puncture.

### Study design

This cross-sectional descriptive study involved 420 blood donors’ serum samples. Samples were gathered randomly from blood donor volunteers in Menoufia University hospitals’ blood banks between January and April 2023. The inclusion criteria included individuals aged 18 and above who volunteered to participate by giving blood and providing informed permission. Individuals with a history of chronic diseases, recent infections, or who refused to participate were excluded from the study. Menoufia Province is a governorate in northern Egypt near the Nile Delta. Its surface area is about 2,543.03 km^2^, with 4,366,000 people in total, as reported in 2018, and its longitude and latitude are 30.52° N and 30.99° E. The governorate is considered one of Egypt’s regions with the highest population densities and is an important center for liver transplantation at the National Liver Institute.

### Sample size estimation

The present sample size was calculated according to Yılmaz et al. (2021)^9^, who revealed 2.3% *T. gondii* IgM seropositivity at alpha error 0.05 and the power of the study 90%; the estimated sample size was 396 participants. Under the following formula,

$${\text{n }} = {\text{Z}}\alpha - {12 } * \, \left( {{\text{pq}}} \right)$$

e2,where n = sample size, z = standard error with the chosen level of confidence (1.96), *p* = proportion detected in the reference study, q = 1 − *p*, and e = acceptable sample error (0.05).

### Questionnaire

A predesigned questionnaire was taken from each participant. It included:

1. Socio-demographic data.
2. Awareness about *T. gondii* infection: was assessed through a series of questions assessing the fundamental understanding of the disease, the transmission routes, hosts, the role of raw meat consumption in transmission, agricultural-related activities and other suggested risk factors, and possible complications of *T. gondii* infection, particularly for pregnant women and persons with weakened immune systems. Through 15 questions that were scored as (2, for correct answer; 1, for incomplete answer; and 0, for wrong answer, with a total score of 30; the good awareness level was at a score of 15 or above while the score less than 15 was considered as poor awareness.
3. Risk factors associated with *T. gondii* infection: including dealing with cats, agricultural environment-related activity, eating or dealing with raw meat as well as hand washing before eating, it also included other data, including blood group type, and previous blood transfusion.

### Blood Sampling

Each person donated three mL of venous blood, centrifuged for five minutes at 3000 rpm to extract the serum and kept at − 20 °C for further laboratory analysis.

### Enzyme-linked immunosorbent assay (ELISA)

Serum samples were transferred to the Parasitology Laboratory, Department of Clinical and Molecular Parasitology, National Liver Institute, Menoufia University, Egypt, to detect *T. gondii -specific* IgM and IgG antibodies. All were analyzed using an ELISA kit that is available commercially (Cat No. SL2055Hu_1 and SL2054Hu-1, SunLong Biotec). The manufacturer’s guidelines were fulfilled for running the analysis. Based on ELISA kits, positive samples were considered at titers above 1 and 3 IU for IgM and IgG, respectively. Negative samples were defined at values below 0.8 and 1 IU for IgM and IgG, respectively. Between the two ranges, a grey zone is reported. The optical density (OD) was measured under a 450 nm wave.

### Venereal infection screening

All samples were tested for HBV surface antigen (HBsAg), anti-HCV antibodies, HIV p24 antigen, anti-HIV antibodies, and anti-*T. pallidum* antibodies. The venereal infection screening was conducted using an immunoassay Cobas e 601 immunoassay analyzer (Roche Diagnostics, Germany), which employs electrochemiluminescence (ELC) technology. The tests used were Elecsys HBSAGII (Cat No. 07251076190), Elecsys AHCVII (Cat No. 06427405190), Elecsys HIV Duo test (Cat No. 07229542190), and Elecsys Syphilis (Cat No. 07251378190), all provided by COBAS (Roche Diagnostics) and performed according to the manufacturer’s instructions.

### Statistical analysis

Categorical and quantitative data were analyzed using SPSS (Statistical Package Software for Social Science) version 20.0 (SPSS Inc., Chicago, IL, USA). The prevalence of *T. gondii* antibodies and positivity to other transfusable venereal infections were assessed through frequency, percentage, and a 95% confidence interval (CI). Comparing positive and negative *T. gondii* antibody groups regarding qualitative variables by chi-squared test and quantitative normally distributed data was tested by student’s t-test. The study employed multivariate binary logistic regression analysis to calculate adjusted odds ratios (ORs) and 95% confidence intervals (CIs) to determine independent risk factors for *T. gondii* infection. A p-value of less than 0.05 determined a statistically significant result.

## Results

### Prevalence of transfusable venereal infections

**Fig. 1:**
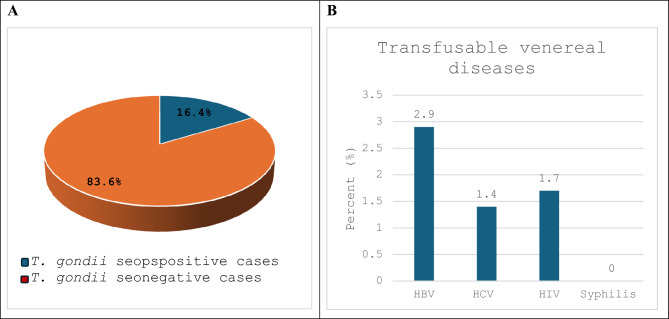
(**A**): Prevalence of *T. gondii* among blood donors (**B**): Prevalence of transfusable venereal diseases.

Regarding the prevalence of transfusable venereal infections, initial screenings (for HBV, HCV, HIV, and syphilis) detected 12 cases (2.9%) positive for HBsAg, six positive cases for anti-HCV antibodies (1.4%), seven cases positive for HIV p24 antigen and anti-HIV antibodies (1.7%), and nonpositive for syphilis (Fig. 1,B).

### Seropositivity of *T. gondii* infection

The ELISA test screened 420 blood samples for *T. gondii-*specific IgG and IgM antibodies. Out of them, 69 (16.4%) blood donors had anti-*T. gondii* antibodies in their sera (IgG, IgM, or both) (Fig. 1, A). Forty-six cases (11%) were IgG-only seropositive, 22 cases (5.2%) were IgM-positive, and one case was positive for both IgG and IgM (0.2%) (Table 1).

**Table 1:** *T. gondii* -specific antibodies among screened blood donors.

	The studied blood donors N = 420	
	Estimates (%)	95% CI
*T. gondii* IgM	22 (5.2%)	3.3–7.8
*T. gondii* IgG	46 (11.0%)	8.1–14.3
*T. gondii* IgM + IgG	1 (0.2%)	0.0–1.3
*T. gondii* Negative Ig	351 (83.6%)	79.7–87

*CI* Confidence interval.

### Demographic characteristics of the studied population

Among the healthy blood donors enlisted in this research, the respondents’ average age was 32.39 ± 10.51 years (with a range of 17–66 years). Ages 21–40 comprised the largest age cohort of blood donors (68.1%). The vast bulk of the subjects (97.1%) were men. Sixty-six-point two percent (66.2%) of the volunteers were highly educated (Table 2).

**Table 2:** Demographic Data, Epidemiological Variables, Awareness, and Blood Type of Participants (N = 420).

Sociodemographic data	The studied blood donors N (%) 420 (100%)
Age (years)	
Mean ± SD	32.39 ± 10.51
Range	17–66
Age groups (years)	
< 20	40 (9.5%)
21–40	286 (68.1%)
41–60	90 (21.4%)
> 60	4 (1.0%)
Sex	
Mal	408 (97.1%)
Female	12 (2.9%)
Residence	
Rural	239 (56.9%)
Urban	181 (43.1%)
Education	
Low level	142 (33.8%)
High level	278 (66.2%)
Risk factors	
Dealing with cat	
Yes	46 (11.0%)
No	374 (89.0%)
Raw meat	
Yes	86 (20.5%)
No	334 (79.5%)
Agriculture	
Yes	72 (17.1%)
No	348 (82.9%)
Blood transfusion	
Yes	22 (5.2%)
No	398 (94.8%)
Awareness	
Good	42 (10.0%)
Poor	378 (90.0%)
Blood grouping	
A^+^	129 (30.7%)
A^−^	11 (2.6%)
B^+^	96 (22.9%)
B^−^	4 (1.0%)
AB^+^	43 (10.2%)
AB^−^	1 (0.2%)
O^+^	123 (29.3%)
O^−^	13 (3.1%)

### Significant risk factors

Sixty-three (15.4%) of male-positive cases and six (50%) of female-positive cases indicated that the female sex was a major risk factor. Also, dealing with cats, eating rand, dealing with row meat, the agricultural environment, poor awareness about *T. gondii* infection, and blood groups were significant risk factors for *T. gondii* infection. Age, residence, educational level, and the presence of other transfusable venereal infections weren’t associated with the *T. gondii* infection (Table 3). There was no significant association between seropositivity for *T. gondii* and venereal infections (Table 4).

**Table 3:** *T. gondii* seropositivity in relation to sociodemographic criteria among the studied blood donors.

	*T. gondii* seropositivity		Test	*P* value
	Seropositive N = 69	Seronegative N = 351		
Age (years)				
Mean ± SD	30.09 ± 9.64	32.84 ± 10.62	1.92	0.09
Range	17–52	17–66		
Age groups (years)				
< 20	9 (22.5%)	31 (77.5%)	2.12	0.55
21–40	47 (16.4%)	239 (83.6%)		
41–60	13 (14.4%)	77 (85.6%)		
> 60	0 (0.0%)	4 (100%)		
Sex				
Male	63 (15.4%)	345 (84.6%)	10.14	0.001*
Female	6 (50.0%)	6 (50.0%)		
Residence				
Rural	37 (15.5%)	202 (84.5%)	0.60	0.32
Urban	32 (17.7%)	149 (82.3%)		
Education				
Low level	25 (17.6%)	117 (82.4%)	0.22	0.64
High level	44 (15.8%)	234 (84.2%)		
Risk factors				
Dealing with cat				
Positive	22 (47.8%)	24 (52.2%)	37.09	< 0.001*
Negative	47 (12.6%)	327 (87.4%)		
Eating raw meat				
Positive	47 (54.6%)	39 (45.3%)	115.08	< 0.001*
Negative	22 (6.6%)	312 (93.4%)		
Agriculture environment				
Positive	26 (36.1%)	46 (63.9%)	24.52	< 0.001*
Negative	43 (12.4%)	305 (87.6%)		
Blood transfusion				
Positive	6 (27.3%)	16 (72.7%)	1.99	0.16
Negative	63 (15.8%)	335 (84.2%)		
Awareness				
Good	15 (35.7%)	27 (64.3%)	12.64	< 0.001*
Poor	54 (14.3%)	324 (85.7%)

**Table 4:** *T. gondii* seropositivity in relation to transfusable venereal diseases among the studied blood donors.

	*T. gondii* seropositivity		Test	*P* value
	Seropositive N = 69	Seronegative N = 351		
HBV				
Positive	3 (25.0%)	9 (75.0%)	0.66	0.43
Negative	66 (16.2%)	342 (83.8%)		
HCV				
Positive	2 (33.3%)	4 (66.7%)	1.27	0.26
Negative	67 (16.2%)	347 (83.8%)		
HIV				
Positive	3 (42.9%)	4 (57.1%)	3.62	0.09
Negative	66 (16.0%)	467 (84.0%)		
Syphilis				
Positive	0 (0.0%)	0 (0.0%)	–	–
Negative	69 (100%)	351 (100%)		
Blood grouping				
A	13 (18.8%)	128 (36.5%)	38.33	< 0.001*
B	4 (5.8%)	97 (27.6%)		
AB	11 (15.9%)	31 (8.8%)		
O	41 (59.4%)	95 (27. %1)		
RhD				
Positive	61 (88.4%)	330 (94.0%)	2.52	0.09
Negative	8 (11.6%)	21 (6.0%)		

* Significant differences.

### Multivariate analysis of independent risk factors associated with *T. gondii* infection

Multivariate regression analysis revealed that female gender, consumption of raw meat, agriculture environment and poor *T. gondii* infection awareness were independent risk factors for *T. gondii* infection with an odds ratio (95% CI): 3.1 (1.81–9.45), 32.62 (13.14–81.0), 4.57 (2.01–10.41), 12.66 (4.53–35.42) for the female gender, consumption of raw meat, agriculture environment, lack of *T. gondii* infection awareness respectively while for ABO grouping with taking B group as a reference, AB and O groups were independent risk groups with odds ratio (95% CI): 3.26 (1.92–7.84) & 4.58 (2.11–11.47) respectively (Table 5).

**Table 5:** Multivariate binary logistic regression analysis for independent risk factors for *T. gondii* infection.

	SE	X^2^	*P* value	Odds ratio	95% CI	
					Lower	Upper
Sex	0.74	15.78	< 0.001*	3.1	1.81	9.45
Dealing with cats	0.42	0.10	0.75	1.15	0.49	2.66
Eating raw meat	0.44	60.34	< 0.001*	32.62	13.14	81.0
Agriculture environment	0.39	14.89	< 0.001*	4.57	2.01	10.41
Awareness	0.49	23.19	< 0.001*	12.66	4.53	35.42
ABO						
B	1.71	15.79	< 0.001*	1	–	–
A				1.22	0.86	6.25
AB				3.26	1.92	7.84
O				4.58	2.11	11.47

*CI* Confidence interval, * significant differences.

## Discussion

Understanding the prevalence of *T. gondii* and venereal infectious pathogens and risk factors among blood donors in Menoufia Province is crucial for public health strategies. This research is an epidemiologic report on seropositivity to *T. gondii* infection among healthy blood donors in Menoufia blood banks, Egypt. Menoufia Governorate had a low prevalence compared to most worldwide studies. In this research, the authors reported a total seroprevalence of 16.4% (95% CI 13–20.3); IgM-positive cases represented 5.5%, posing a risk of transmitting the infection to blood recipients. By integrating molecular approaches, supplementary serological markers, and direct proof of parasitemia, the hypothesis can be substantially reinforced, leading to a more thorough evaluation of the risk of *T. gondii* infection by blood transfusion, which is undertaken currently in epidemiological national research funded by STDF aiming to complete the current research.

Globally, according to estimates by Foroutan-Rad et al.^10^, *T. gondii* infection affects 33% of blood donors worldwide, with rates highest in Africa (46%) and lowest in Asia (29%)^10^. The prevalence rate varies by nation: 6.26% in China^11^, 9.3% in Taiwan^12^, 19.66% in India^13^, 20.5% in Serbia^14^, 25.6% in Turkey^9^, 36% in Portugal^15^, 48.1% in Brazil^16^, and 67.92% in Côte d’Ivoire^17^.

In other African countries, the seroprevalence among tested blood donors was 44.4% in South-West and Central-East Tunisia^18^ and 47.7% in Sidi Bel Abbès, West Algeria^19^. The difference in serological methods used across studies is probably the main factor in the difference in reported prevalence of *T. gondii* infection among different nations.

Compared with previous findings from other Egyptian governorates, the current seroprevalence rates are consistent with those from El-Wadi El Gadded, which had the lowest incidence between 1 and 25%^20^. Earlier studies reported a prevalence between 33.7 and 67.4% of healthy Egyptian blood donors had antibodies to *T. gondii* infection, comparable to a range of 3–42.5% in the general Egyptian population. Increased seropositivity was seen. in the Lower Egypt bordering governorates of Sharqia and Qalyoubia (38.8% and 27.5% respectively), as well as in the rural Upper Egypt governorate of Beni-Suef (35.2%)^21^. Cairo also had high infection rates (between 30 and 42.5%)^21^. The studied group’s higher level of illness knowledge is probably the reason for the reduced infection prevalence when compared to estimates from throughout the world. These differences point to possible socioeconomic and geographic variables affecting *T. gondii* exposure in Egypt.

Multivariate regression analysis displayed that contact with cats, consuming raw or undercooked meat, and having agricultural pursuits are significant risk factors for *T. gondii* seropositivity, demonstrating that both infection routes—ingesting oocysts (soil contamination, contaminated water, and contaminated raw food e.g. salads, vegetables) and tissue cysts found in undercooked meat (a foodborne transmission)—showed up among the blood donors with different educational levels. These findings are supported by earlier studies^9,22^. From their results, domestic cats may be related to the exposure of the individuals included in the study to *T. gondii*. However, it is worth noting that direct contact with cats does not guarantee transmission of the parasite since *T. gondii* oocysts are eliminated as non-infective. In contrast to the present findings, El-Deeb and their alleles^23^ found no statistically significant association between seropositivity concerning contact with domestic cats and meat consumption in Menoufia, Egypt. However, contact with soil was a considerable risk factor, which could be explained by the prevalence of domestic and stray cats, both more susceptible to parasites^23^.

Likewise, in the research done by Mahmoudvand et al.^22^, the prevalence of *T. gondii* infection in the current study was significantly higher in female donors (95%CI 1.71–17.52) despite the limited number of female participants in our study compared to male donors. Mahmoudvand et al.^22^, attributed this disparity to the female daily exposure to more tissue cysts and oocysts. Handling raw meat and gardening are cultural practices and household activities that may expose women to greater levels of *T. gondii*. Therefore, validating these findings using a more extensive sample size is necessary. These results were not supported by Hosseini and his/ her colleagues^24^, who did not find gender a significant risk factor.

Seropositivity in this research was higher in rural areas (53.6%) than in urban areas (46.4%); however, the difference was insignificant. This finding contrasts with those reported by some authors^22,24^. They hypothesized in their research that the overabundance of cats, inadequate sanitation of the environment, and lax hygiene standards might cause this difference.

The ABO phenotype and RhD antigen were previously associated with pathogenic protozoa of the phylum Apicomplexa. The protective effect of type O blood against severe malaria has been observed, possibly explaining the high prevalence of type O in regions where *Plasmodium falciparum* is endemic^25^.

Our current research discovered that blood donors carrying the type O blood group had the highest incidence of *T. gondii* infection and were riskier, with a significant difference between *T. gondii* and (*P* < 0.001), which is equivalent to the findings reported previously in northern Egypt^26^ but different from those reported in Iran, where they found blood group B carriers more susceptible to infection with *T. gondii* infection^24^. Following the findings of Hosseini et al. research, the level of disease between Rh-positive and negative samples was not different^24^. Despite the association our study found between the blood group and seropositivity, this does not prove that the two are causally related to the onset of illness. Our study’s findings should be seen as preliminary and need more investigation in follow-up studies.

Most positive cases ranged from 21 to 40 years; however, age was not a significant risk factor in our univariate analysis. In the same vein, research done in Ardabil Province, northwestern Iran, demonstrated that most positive cases were aged 31–40 with no significant difference^27^. Unlike the current finding, other authors found that age substantially contributes to infection. Their conclusion was attributed to the cumulative effect of being exposed to the parasite over time^14^.

This research showed a higher prevalence of HBsAg (2.9%), followed by HIV and HCV (1.7% and 1.4%, respectively). Syphilis cases were absent among the studied population. The higher percent of HBsAg compared to other screened transfusion-transmissible infections was consistent with similar reports from a study among blood donors in Bahir Dar, North West, Ethiopia, where HBV was prevalent in 2.8% of cases, followed by HIV and HCV^28^.

Co-infections can worsen the symptoms of some infectious disorders. It can modulate immune responses, exacerbate disease severity, and increase inflammatory cytokines. While this study did not find a substantial prevalence of co-infection between *T. gondii* and the viral agents tested (HBV, HIV, and HCV), other research suggests that these pathogens may interact. In Egypt, for example, *T. gondii* co-infection with HBV and HCV was reported^29^. Furthermore, HIV infection may impair the immune system, increasing the risk of reactivating latent *T. gondii* infection^8,11^. *T. gondii* co-infection with certain viruses must be addressed to prevent, detect, and cure infections. It needs further examination and research.

## Conclusion and recommendations

This cross-sectional research investigated the seroprevalence of *T. gondii* and common transfusable venereal infections across healthy blood donors in Egypt’s central Menoufia blood banks. According to this study, the governorate of Menoufia had a low incidence of *T. gondii* infection among blood donors.

Therefore, testing for *T. gondii* infection is required in blood donors to prevent potentially fatal outcomes for blood receivers. Building programs for health education are also required as a suitable strategy for preventing diseases.

### Value-added of this research

We addressed the seroprevalence of *T. gondii* in the studied population, which provides a step for further studies and implementation research on a larger scale to test preventive strategies in the future.

## Data Availability

This article encompasses all data that was generated or evaluated.The corresponding author will provide any additional inquiries.

## Abbreviations

CBC: Complete blood picture
HBV: Hepatitis B virus
*T. pallidum*: *Treponema pallidum*
HCV: Hepatitis C virus
HIV: Human immunodeficiency virus
ELISA: Enzyme-linked immunosorbent assay
IgG: Immunoglobulin G
IgM: Immunoglobulin M

## References

[1] Sultan, S. *et al.* Trends of venereal infections among healthy blood donors at Karachi. **19**, 192–196 (2016). 26923891

[2] Pozio, E. How globalization and climate change could affect foodborne parasites. *Exp. Parasitol.* **208**, 107807 (2020). 31751558 10.1016/j.exppara.2019.107807

[3] Cantey, P. T., Montgomery, S. P. & Straily, A. Neglected parasitic infections: What family physicians need to know—A CDC update. *Am. Fam. Physician.* **104**(3), 277–287 (2021). 34523888 PMC9096899

[4] Yan, C., Liang, L.-J., Zheng, K.-Y. & Zhu, X.-Q. Impact of environmental factors on the emergence, transmission and distribution of *Toxoplasma gondii*. *Parasit. Vectors* **9**, 137 (2016). 26965989 10.1186/s13071-016-1432-6 PMC4785633

[5] Shapiro, K. *et al.* Environmental transmission of *Toxoplasma gondii*: Oocysts in water, soil and food. *Food waterborne Parasitol.* **15**, e00049 (2019). 32095620 10.1016/j.fawpar.2019.e00049 PMC7033973

[6] Elhence, P., Agarwal, P., Prasad, K. N. & Chaudhary, R. K. Seroprevalence of *Toxoplasma gondii* antibodies in North Indian blood donors: Implications for transfusion transmissible toxoplasmosis. *Transfus. Apher. Sci. Off. J. World Apher Assoc. Off. J. Eur. Soc. Haemapheresis* **43**, 37–40 (2010). 10.1016/j.transci.2010.05.004 20605111

[7] Arefkhah, N. *et al.* Molecular genotyping and serological evaluation of *Toxoplasma gondii* in mothers and their spontaneous aborted fetuses in Southwest of Iran. *Comp. Immunol. Microbiol. Infect. Dis.* **66**, 101342 (2019). 31437675 10.1016/j.cimid.2019.101342

[8] Bazmjoo, A. *et al.**Toxoplasma gondii*, HBV, and HCV co- infection and their correlation with CD4 cells among Iranian HIV-positive patients. *Immunity. Inflamm. Dis.* **11**, 794 (2023). 10.1002/iid3.794 PMC9947625 36840494

[9] Yılmaz, A., Yazıcı, E. & Turk, C. Assessment of seroprevalence of *Toxoplasma gondii* in blood donors applied to the blood center of Gazi University Hospital. *Iran. J. Microbiol.* **13**, 243–247 (2021). 34540160 10.18502/ijm.v13i2.5986 PMC8408027

[10] Foroutan-Rad, M. *et al.* Toxoplasmosis in blood donors: A systematic review and meta-analysis. *Transfus. Med. Rev.* **30**, 116–122 (2016). 27145927 10.1016/j.tmrv.2016.03.002

[11] Wang, T. *et al.* Seroprevalence of *Toxoplasma gondii* infection in blood donors in mainland China: A systematic review and meta-analysis. *Parasite*10.1051/parasite/2018037 (2018). 30040610 10.1051/parasite/2018037 PMC6057739

[12] Chiang, T.-Y. *et al.* Seroepidemiology of *Toxoplasma gondii* infection among healthy blood donors in Taiwan. *PLoS One* **7**, e48139 (2012). 23133557 10.1371/journal.pone.0048139 PMC3484999

[13] Stephen, S., Pradeep, J., Anitharaj, V. & Janarthanam, V. Seroprevalence of toxoplasmosis in voluntary blood donors of Puducherry and surrounding districts of Tamil Nadu. *J. Parasit. Dis.* **41**, 1158–1161 (2017). 29114158 10.1007/s12639-017-0949-8 PMC5660049

[14] Stopić, M. *et al.* Epidemiology of toxoplasmosis in SERBIA: A cross-sectional study on blood donors. *Microorganisms* **10**, 492 (2022). 35336068 10.3390/microorganisms10030492 PMC8948843

[15] Rodrigues, F. T. *et al.* Seroepidemiology of *Toxoplasma gondii* in blood donors in Portugal. *Transfus. Apher. Sci.* **59**, 102777 (2020). 32487512 10.1016/j.transci.2020.102777

[16] Nakashima, F. *et al.* Serum IgG anti-*Toxoplasma gondii* antibody concentrations do not correlate nested PCR results in blood donors. *Front. Cell. Infect. Microbiol.* **9**, 461 (2019). 31993377 10.3389/fcimb.2019.00461 PMC6970978

[17] Siransy, L. *et al.* Immunity status of blood donors regarding *Toxoplasma gondii* infection in a Low-Income district of Abidjan, Côte d’Ivoire. *West Africa. J. Immunol. Res.* **2016**, 6830895 (2016). 27795962 10.1155/2016/6830895 PMC5066025

[18] Lachkhem, A. *et al.* Seroprevalence of *Toxoplasma gondii* among healthy blood donors in two locations in Tunisia and associated risk factors. *Parasite* **27**, 51 (2020). 32955429 10.1051/parasite/2020049 PMC7504876

[19] Belkacemi, M. & Heddi, B. Toxoplasmosis immunity status of blood donors in Sidi Bel Abbès. *West Algeria. Cureus* **14**, e28826 (2022). 36225427 10.7759/cureus.28826 PMC9535615

[20] Bayoumy, A., Ibrahim, W. L. F., Abou El Nour, B. M. & Said, A. A. A. The parasitic profile among school children in El-wadi El-gadded governorate. *Egypt. J. Egypt. Soc. Parasitol.* **46**, 605–612 (2016). 30230757

[21] Abou Elez, R. M. M., Hassanen, E. A. A., Tolba, H. M. N. & Elsohaby, I. Seroprevalence and risk factors associated with *Toxoplasma gondii* infection in domestic rabbits and humans. *Vet. Parasitol. Reg. Stud. Rep.* **8**, 133–137 (2017). 10.1016/j.vprsr.2017.02.009 31014631

[22] Mahmoudvand, H. *et al.* Seroprevalence and risk factors of *Toxoplasma gondii* infection among healthy blood donors in south-east of Iran. *Parasite Immunol.* **37**, 362–367 (2015). 25891186 10.1111/pim.12198

[23] El Deeb, H. K., Salah-Eldin, H., Khodeer, S. & Allah, A. A. Prevalence of *Toxoplasma gondii* infection in antenatal population in Menoufia governorate. *Egypt. Acta Trop.* **124**, 185–191 (2012). 22921952 10.1016/j.actatropica.2012.08.005

[24] Hosseini, S. A. *et al.* A serological investigation and genotyping of *Toxoplasma gondii* among Iranian blood donors indicates threat to health of blood recipients. *Transfus. Apher. Sci.* **59**, 102723 (2020). 31948918 10.1016/j.transci.2020.102723

[25] Jajosky, R. P. *et al.* The impact of ABO and RhD blood types on *Babesia microti* infection. *PLoS Negl. Trop. Dis.* **17**, e0011060 (2023). 36696414 10.1371/journal.pntd.0011060 PMC9901808

[26] Elsheikha, H. M. *et al.* Seroprevalence of and risk factors for *Toxoplasma gondii* antibodies among asymptomatic blood donors in Egypt. *Parasitol. Res.* **104**, 1471–1476 (2009). 19198880 10.1007/s00436-009-1350-z

[27] Asfaram, S. *et al.* High occurrence of *Toxoplasma gondii* infection among blood donors in Ardabil Province as main focus of zoonotic visceral leishmaniosis, northwestern Iran. *Ann. Parasitol.* **67**, 611–617 (2021). 35247301 10.17420/ap6704.377

[28] Legese, B. *et al.* Association of ABO and rhesus blood types with transfusion-transmitted infections (TTIs) among apparently healthy blood donors at Bahir Dar blood bank, Bahir Dar, North West, Ethiopia: A retrospective cross-sectional study. *J. Blood Med.* **13**, 581–587 (2022). 36238231 10.2147/JBM.S374851 PMC9552785

[29] El-sayed, N. M., Ramadan, M. E. & Ramadan, M. E. *Toxoplasma gondii* infection and chronic liver diseases: Evidence of an association. *Tropical Med. Infect. Dis.*10.3390/tropicalmed1010007 (2016). 10.3390/tropicalmed1010007 PMC6082049 30270858

